# Supplementary material for: Identification of Microorganisms from Several Surfaces by MALDI-TOF MS: P. aeruginosa Is Leading in Biofilm Formation
Source: Microorganisms. 2021 May 4;9(5):992. doi: 10.3390/microorganisms9050992 (PMC8147854; doi:10.3390/microorganisms9050992)
Supplement: Supplementary file 1 [file microorganisms-09-00992-s001.zip › microorganisms-1204190-supplementary.pdf]

Supplementary

Table S1. Overview of monoculture biofilm formation behavior of different bacterial to the Congo Red Agar (CRA) method... CRA were visually scored by using a scoring board shown in Supp. 1.

Bacteria	CRA
P. aeruginosa	None
P. mendocina	None
P. oleovorans	None
P. pseudoalcaligenes	None
P. stutzeri	None
S. arlettae	Moderate
S. capitis	High
S. epidermidis	High
S. hominis	High
S. haemolyticus	High
S. saprophyticus	High
E. coli	None
P. putida	None

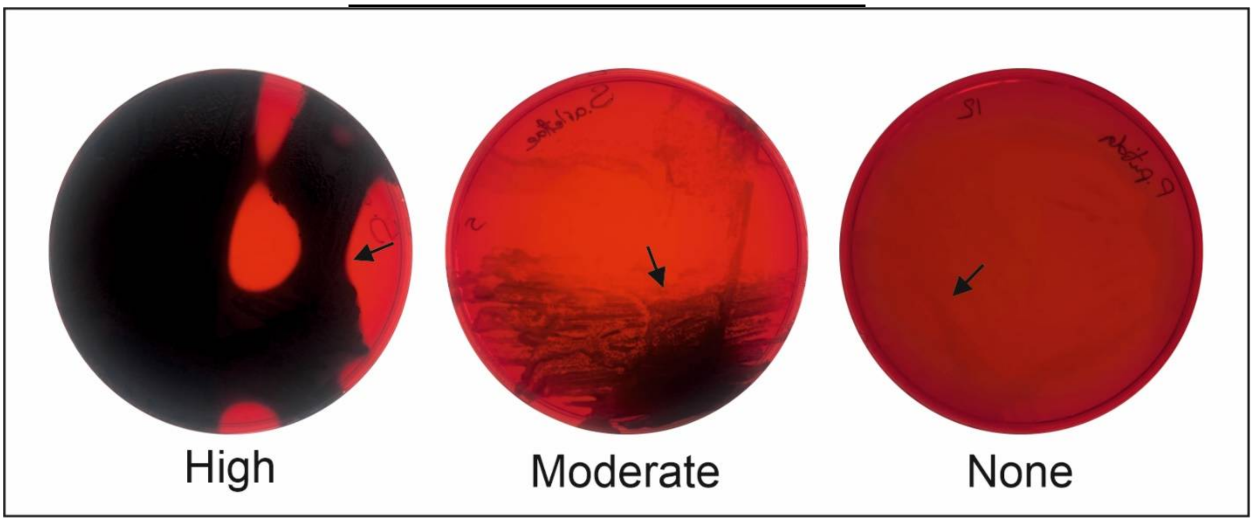

Figure S1. Examples of the quantification of Biofilm intensity for Congo Red Agar, left plate (*S. epidermidis*) represent biofilm producing bacteria as dark black colonies (marked by arrows), middle

plate (*S. arlettae*) shows moderate biofilm potential by light dark colonies (marked by arrows) and right plate (*P. putida*) reveals no biofilm forming bacteria, note no dark colonies.

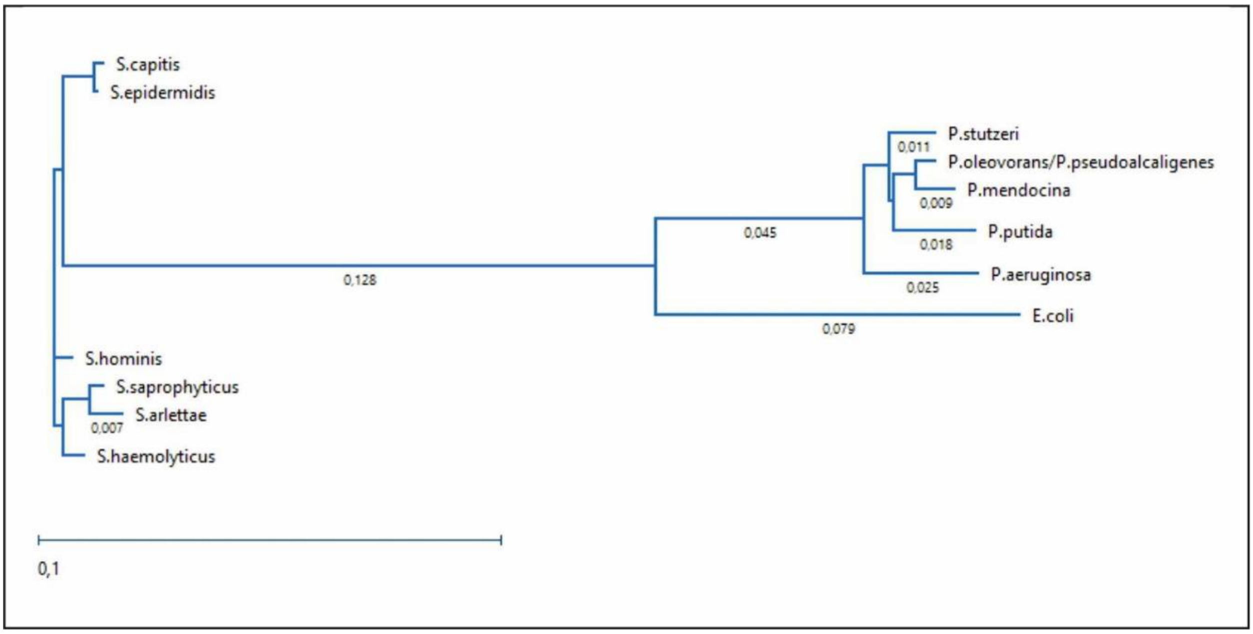

Figure S2. Phylogenic tree of 16S rRNA sequences of Staphylococci, Pseudomonads, and Escherichia coli. To show the homology between the 16S rRNA sequences between the species we performed a multiple sequence alignment of published 16S rRNA sequences for Staphylococci and Pseudomonads, followed by a neighbor joining phylogenic tree tool (DNASTAR).
